# Introductory Lectures

**Published:** 1848-01

**Authors:** 


### INTRODUCTORY LECTURES.

Two introductory lectures of the present season have been placed on our table—one delivered by Prof. Drake to the students in the medical department of the University of Louisville; the other by Prof. Lawson to the class in the Ohio Medical College. Professor Drake entitles his lecture "Strictures on some of the defects and infirmities of intellectual and moral character in students of medicine." In the discussion, he evinces that mastery of his subject which nearly thirty years' experience as a teacher might be expected to give. The lecture is practical and to the point. It deals plainly with "the defects and infirmities" of medical students, and indicates the method by which they are to be countervailed and cured. The lecture closes with the following patriotic thoughts:

"In the fourth place, our students do not propose to themselves, that they will strive to enlarge the boundaries of our science. But why should they not? The American mind is not inferior in strength or invention to that of Europe. In the science of government—in jurisprudence—in eloquence—in war—in agriculture—in the fine and useful arts, it has shown itself quite equal, often superior, to the European mind. Why is it, then, that *we* continue to look, almost exclusively, to our medical brethren on the other side of the ocean, and not to ourselves for discoveries and improvements? France, Germany, England, and Ireland, are respectively cultivating the profession, as if the duty of enlarging its boundaries, rested on one only. The physicians of those several kingdoms, do not turn to each other with an eye of dependence, but of emulation. In this country, the reverse prevails. To acquire a tolerable knowledge of what is done abroad, too often constitutes the sum total of the ambition of the best minds among us, while hundreds do not even aspire to that. It is time that we threw of the shackles of colonial dependence, and assumed a higher position. Once, like callow nestlings, we of necessity swallowed whatever our European nursing mothers dropped into our gaping throats; but be are no longer unfledged, and should spread out our growing pinions, and begin a life of independent activity. This resolution once taken, success would not be doubtful. But who should take it? Those who are now in, or those who are aspiring to the profession? I answer, *all*, but especially the latter—you and your fellow students throughout the Union;—for the old are too inert, the middle-aged too indifferent, and a majority of both classes too little prepared by early discipline or lofty aspirations to enter on a career of glory. Our hopes, therefore, must rest on you more than your preceptors and seniors. Would to God, my young friends, that I could kindle in your hearts a flame of patriotic ambition, so intense as to burn up every germ of *servile* dependence on the physicians of the old world! Would that I could fill your imaginations with bright and beautiful visions of the future! But should I do so, remember that the most glorious conceptions will not, of themselves, secure the objects which they embody. The conceptions of mind which tell on the progress of an individual, a class, or a nation, comprehend both causes and effects. The little child as eagerly puts forth its hands to pluck the flower which hangs beyond its reach, as that which blossoms within its grasp. Experience tends to correct this constitutional error of infancy; but in many minds the correction is so slow and imperfect, that the man, in this strait of character, remains a child; and while he indulges in captivating visions of excellence, which cost no effort, neglects the labors which alone can convert them into living realities. They who do not rise above this inferiority of our nature, will do nothing towards emancipating us from our unequal dependence on Europe. They who surmount it, will contribute to the foundation of an American profession. To this great and brilliant enterprise your *alma mater* now calls you—every one, the humblest not less than the highest, for all may contribute something. How happy would she be, to see every heart here, and in all the sister schools of the Continent, quickened into life, and filled with a moral courage equal to the task! Then, the question with the young aspiant would no longer be—What saith the seer of other countries? but of our own, our beloved native land: then, improvement would roll onward, like a mighty river—deep and fertilizing as the Mississirpi—transparent and uniform as the St. Lawrence! The fame of its waters would spread to other lands; and many of their young men would come and drink. American students as they traversed the ocean for the East, would then greet European students on their way to the West; and on reaching the schools of the old world, would feel no humiliation; but in the pride of a glowing patriotism, would be able to exclaim to those around them—'If we have sought the banks of the Thames and the Seine, your young men are treading those of the Delaware, the Hudson, and the Ohio.'"

Professor Lawson's lecture is written in a fervid style, and abounds in sentiments calculated to fire the ambition of his pupils. It speaks well for the good taste of his class that they asked it for publication. On the cover of the lecture is the prospectus of the Western Lancet, which hereafter is to be edited by Drs. Lawson and Harrison, and published at Cincinnati.

